# Supplementary material for: Identification of photoperiod-induced specific miRNAs in the adrenal glands of Sunite sheep (Ovis aries)
Source: Front Vet Sci. 2022 Jul 22;9:888207. doi: 10.3389/fvets.2022.888207 (PMC9354845; doi:10.3389/fvets.2022.888207)
Supplement: Supplementary file 1 [file Data_Sheet_1.ZIP › Supplementary file/miRDeep2 file.zip!/miRDeep2 file/Know_Structure/LP42A1/oar-mir-16b.pdf]

miRBase precursor : oar-mir-16b
Total read count : 51532
oar-miR-16b read count : 51469
remaining reads : 63

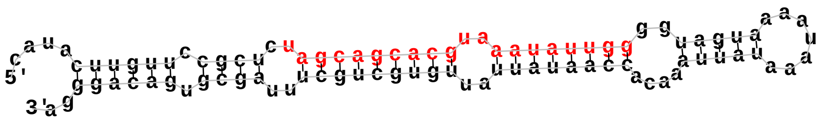

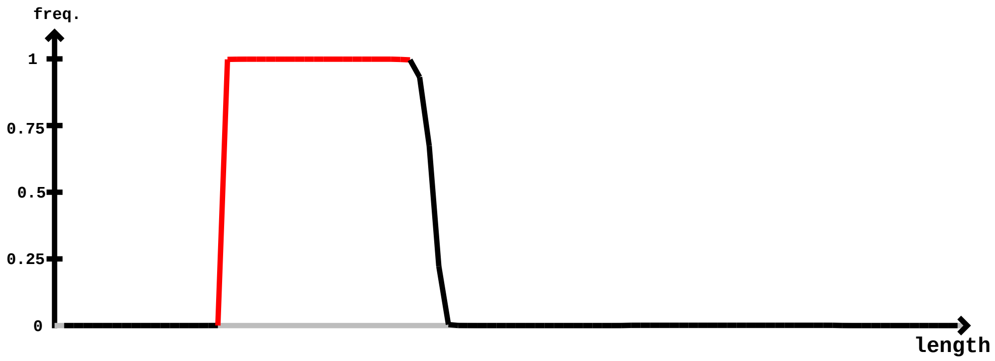


```
                oar-miR-16b
5'- cauacuuguuccgcucuagcagcacguaaauauugggguaguaaaauaaauauuaaacaccaauauuauugugcugcuuuagcgugacaggga   -3'    exp
    ....((((((.((((..(((((((((..((((((((..(((((.......)))))....))))))))..)))))))))..)))).))))))..    reads   mm   sample
    ............gcucuagcagcacguaaauauugggg.......................................................    1       0    seq
    ............gcucuagcagcacguaaauauuggggu......................................................    1       0    seq
    ..............ucuagcagcacguaaauauugg.........................................................    1       0    seq
    ...............cuagcagcacguaaauauugg.........................................................    4       0    seq
    ...............cuagcagcacguaaauauuggg........................................................    1       0    seq
    ...............cuagcagcacguaaauauugggg.......................................................    1       0    seq
    ...............cuagcagcacguaaauauuggggu......................................................    2       0    seq
    ................uagcagcacguaaauau............................................................    2       0    seq
    ................uagcagcacguaaauauu...........................................................    52      0    seq
    ................uagcagcacguaaauauug..........................................................    86      0    seq
    ................uagcagcacguaaauauugg.........................................................    3320    0    seq
    ................uagcagcacguaaauauuggg........................................................    1141    0    seq
    ................uagcagcacguaaauauuggA........................................................    12145   1    seq
    ................uagcagcacguaaauauuggAg.......................................................    20828   1    seq
    ................uagcagcacguaaauauugggg.......................................................    2416    0    seq
    ................uagcagcacguaaauauuggAgu......................................................    7078    1    seq
    ................uagcagcacguaaauauuggggu......................................................    4160    0    seq
    ................uagcagcacguaaauauuggggua.....................................................    182     0    seq
    ................uagcagcacguaaauauugggguag....................................................    10      0    seq
    ................uagcagcacguaaauauugggguagu...................................................    5       0    seq
    .................agcagcacguaaauauugg.........................................................    2       0    seq
    .................agcagcacguaaauauuggA........................................................    4       1    seq
    .................agcagcacguaaauauuggg........................................................    2       0    seq
    .................agcagcacguaaauauuggAg.......................................................    16      1    seq
    .................agcagcacguaaauauugggg.......................................................    1       0    seq
    .................agcagcacguaaauauuggAgu......................................................    2       1    seq
    .................agcagcacguaaauauuggggu......................................................    3       0    seq
    ..................gcagcacguaaauauuggA........................................................    3       1    seq
    ..................gcagcacguaaauauuggAg.......................................................    4       1    seq
    ..................gcagcacguaaauauuggAgu......................................................    1       1    seq
    ..................gcagcacguaaauauuggggu......................................................    2       0    seq
    .........................................................caccaauauuauugugcugcuu..............    1       0    seq
    .........................................................caccaauauuauugugcugcuuu.............    5       0    seq
    ..........................................................accaauauuauugugcugcuu..............    4       0    seq
    ..........................................................accaauauuauugugcugcuuu.............    33      0    seq
```

oar-miR-16b

```
cauacuuguuccgcucuagcagcacguaaauauuggguaguaaaauaaauauuaaacaccaauauuauugugcugcuuuagcgugacaggga
.........................................................accaauauuauugugcugcuuua............    6          0          seq
..........................................................ccaauauuauugugcugcuuu.............    4          0          seq
..........................................................ccaauauuauugugcugcuuua............    3          0          seq
```
